# Comparative Study of the Peptide Profiles of A1/A2 and A2/A2 Yogurts during *In Vitro* Gastrointestinal Digestion

**DOI:** 10.1021/acs.jafc.5c15659

**Published:** 2026-03-03

**Authors:** Laís Firmino, Débora Parra Baptista, Kívea Kássia de Paiva e Silva, Orlando Célio Campovilla, Flavia Maria Netto, Mirna Lúcia Gigante

**Affiliations:** Department of Food Engineering and Technology, School of Food Engineering, 28132 Universidade Estadual de Campinas, UNICAMP, 13083-862, Campinas, SP Brazil

**Keywords:** peptides, proteolysis, β-casein, bioaccessibility, LC-MS/MS

## Abstract

The production of A2 milk and its use in dairy products processing are driven by potential health benefits related to the absence or reduced release of β-casomorphin-7, an opioid peptide generated from β-casein hydrolysis during digestion. Although the release of higher concentrations of β-casomorphin-7 has been consistently reported following the digestion of isolated A1 β-casein and milk containing A1 β-casein, the impact of complex dairy matrices on this peptide release remains underexplored. This study evaluated the peptide profiles of yogurts produced from milks with different β-casein genotypes (A1A2 and A2A2) during *in vitro* gastrointestinal digestion. The release of β-casomorphin-7 was observed in both products’ digestion, but differences in peptide profiles were observed along the β-casein sequence, extending beyond the site of the A1/A2 amino acid substitution.

## Graphical abstract

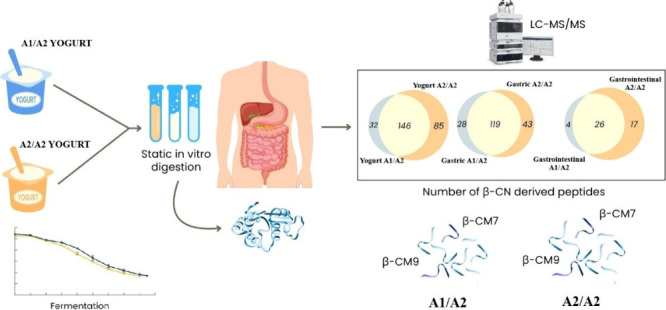

## 1 Introduction

Milk proteins are classified into two groups based on their solubility at pH 4.6. One group consists of proteins that precipitate when milk is acidified to pH 4.6 (isoelectric point) at 20 °C. These proteins, known as caseins, represent approximately 80% of the total milk protein content. The other group, known as whey proteins, includes proteins that remain soluble under these conditions.^1,2^

Caseins exist in milk as colloidal structures known as casein micelles, which comprise the following fractions: α_s1_ (12–15 g/L), α_s2_ (3–4 g/L), β (9–11 g/L), and κ-casein (2–4 g/L).^1^ The four casein fractions exhibit microheterogeneity, which arises from five main factors: differences in phosphorylation, the number of disulfide bonds, hydrolysis of primary casein by plasmin, variations in glycosylation, and genetic polymorphism.^3^ There are eight genetic variants of α_s1_-casein (A, B, C, D, E, F, G, and H), four variants of α_s2_-casein (A, B, C, and D), 12 variants of β-casein (A1, A2, A3, B, C, D, E, F, G, H1, H2, and I), and 11 variants of κ-casein (A, B, C, D, E, F1, F2, G1, G2, H, I, and J).^1^

Several important technological properties of milk proteins, such as coagulation properties essential for producing dairy products like cheese and yogurt, have been associated with the composition of different genetic variants in milk, and significant research is ongoing on this subject.^4–6^ β-casein (β-CN) accounts for approximately 45% of the total caseins in bovine milk, and the genetic variants A1 and A2 are the most abundant in cattle herds.^1,7^ The production of the β-CN genetic variant is determined by a pair of codominant alleles: cows with the A2A2 genotype produce only β-CN A2, those with the A1A2 genotype produce both β-CN A1 and A2, and cows with the A1A1 genotype produce only β-CN A1. Milk that contains only β-CN A2 is referred to as A2 milk, whereas milk containing either only β-CN A1 or both β-CN A1 and β-CN A2 is classified as A1 milk.^8,9^

Dairy proteins are recognized for their positive association with health, as they are considered the primary sources of bioactive peptides.^10,11^ However, consumption of milk containing β-CN A1 may release significant amounts of a specific opioid peptide, β-casomorphin-7 (β-CM7), which is suggested to be linked with the development of some diseases.^12,13^

The β-CN genetic variants A1 and A2 differ only in the amino acid residue at position 67 of the amino acid sequence of this protein, which contains 209 amino acid residues. In β-CN A1, the amino acid at position 67 is Histidine, while in β-CN A2, it is Proline.^1,7^ This difference may affect the hydrolysis of these proteins by digestive enzymes and the release of peptides during digestion. According to an *in vitro* study by Jinsmaa and Yoshikawa,^14^ the formation of the peptide β-CM7 (residues 60–66, YPFPGPI) depends on the β-casein genetic variant, since the hydrolysis of the 66–67 peptide bond, required for β-CM7 release, occurs only when histidine is present at position 67, because this bond is more susceptible to cleavage by pancreatic elastase. In contrast, the presence of proline at this position prevents hydrolysis.

In recent decades, studies have reported the release of β-CM7 following simulated gastrointestinal digestion of both β-CN A1 and A2, with the A2 variant showing significantly lower levels or even the absence of this peptide. Milk with the A2A2 genotype may release up to 4-fold less β-CM7 than milk containing β-CN A1.^15,16^ Asledottir et al.^16^ and Duarte-Vázquez et al.^17^ observed higher β-CM7 release from A1 milk compared to A2 milk after *in vitro* digestion. Similarly, Asledottir et al.,^18^ using *ex vivo* digestion, demonstrated that β-CN A1 released 3-fold more β-CM7 per gram of protein than β-CN A2. Ul Haq et al.^19^ used β-CN isolated from A1A1, A1A2, and A2A2 milk and found that, after simulated digestion, the A1A1 sample released three times more β-CM7 than the A1A2 sample, while this peptide was not detected in A2A2. More recently, Aitchison et al.^21^ analyzed isolated β-CN A1 and A2 and reported the presence of β-CM7 in both, with a predominance in A1. Additionally, they observed the release of β-CM9, which was more abundant in β-CN A2.

Despite the growing interest in the physiological effects of bioactive peptides derived from dairy proteins, the identification of peptides released during digestion, particularly β-casomorphins, remains limited and underexplored in dairy products. The absence of such studies limits a more accurate assessment of the release of opioid peptides under realistic conditions and their potential implications for human health. Thus, the aim of this study was to compare the peptide profiles of yogurts produced with A1/A2 and A2/A2 milk, before and after *in vitro* digestion.

## 2 MATERIAL AND METHODS

Semiskimmed Ultra-High Temperature (UHT) A2 milk (Piracanjuba, Brazil; batches 3198A2-8), designated by the company as A2 milk and mandatorily certified by the Brazilian Confederation of Agriculture and Livestock (CNA) as originating from homozygous A2 animals (A2A2 genotype), was referred to in this study as A2/A2 milk. Conventional semiskimmed UHT milk from the same brand (Piracanjuba, Brazil; batches 3209A2-9), which contains β-CN A1 and β-CN A2 in unknown proportions, was designated in this study as A1/A2 milk. Both products were purchased from local stores in Campinas, São Paulo, Brazil. The conformity and purity of the milks used in the study were evaluated by using a rapid test (A2MilkTest, Scienco Biotech), validated by Albiero et al. (2024). The test allows the detection of β-CN A1 above an approximate threshold of 5%. Thus, the test confirmed the conformity and purity of the milk marketed as A2, whereas the conventional milk indicated the presence of β-CN A1 without the identification of its proportion.

The milk samples were analyzed for total solids by oven drying at 105 °C, ash content by incineration in a muffle furnace at 550 °C, fat content after extraction in Mojonnier flasks, and total nitrogen using the micro-Kjeldahl method, according to official methodologies.^22^ Protein content was calculated by multiplying the total nitrogen content by 6.38. Carbohydrate content was calculated by the difference between the total solid content and the other solid constituents. All analyses were performed in triplicate.

### 2.1 Yogurts Production

For yogurt production, 1.0 L volumes of milk were heated to 45 ± 0.5 °C and inoculated (2.5% v/v) with a mixed lactic culture consisting of *Streptococcus thermophilus* and *Lactobacillus delbrueckii* subsp. *bulgaricus* (YF-L812, CHR Hansen, Valinhos, SP, Brazil), previously activated (45 °C/4 h) in the respective A1 and A2 milks. After inoculation, part of the milk was incubated in an incubator (model AC62/1, AC Cientfica, Piracicaba, SP, Brazil) and maintained at 45 ± 0.5 °C. To monitor acidification, 30 mL aliquots were transferred to screw-cap tubes and placed in a water bath (45 ± 0.5 °C). The pH was measured every 30 min, and fermentation was stopped by cooling (4.0 ± 1.0 °C) when the pH reached 4.8 ± 0.05. On the following day, after gel stabilization, the gels were broken using a glass rod to obtain stirred yogurts, which were stored under refrigeration (4.0 ± 1.0 °C) until digestion. The acidification rate was determined as the pH variation over a 60 min interval (dpH/dt), expressed as pH/min. The process was carried out in triplicate by using milk from the same batch.

### 2.2 In Vitro Simulation of Gastrointestinal Digestion of Yogurts

The *in vitro* simulation of yogurt digestion was performed 8 days after production, following the static protocol proposed by the INFOGEST method.^23^ Briefly, for the oral phase, 5 g of yogurt were mixed with 4 mL of simulated salivary fluid, 25 μL of 0.3 M CaCl_2_, and 975 μL of ultrapure water. The mixture was rapidly mixed and incubated for 2 min at 37 °C. For the gastric phase, 8 mL of simulated gastric fluid, 5 μL of 0.3 M CaCl_2_, and 0.5 mL of pepsin solution (Sigma-Aldrich, P6887, 2000 U mL^–1^) were added. The pH was adjusted to 3.0 using 5 M HCl and water was then added to adjust the total volume of added fluids during gastric digestion to 20 mL. The solution was then incubated at 37 °C for 2 h under agitation. For the intestinal phase, 8 mL of simulated intestinal fluid, 3 mL of bile solution, 40 μL of 0.3 M CaCl_2_, and 5 mL of pancreatin solution (Sigma-Aldrich, P7545, 100 U mL^–1^) were added. The pH was adjusted to 7.0 with 5 M NaOH and water was added to bring the total volume of fluids added during intestinal digestion to 40 mL. The mixture was incubated at 37 °C for 2 h under agitation.

To analyze peptides formed during gastric and gastrointestinal digestion, two independent experiments were conducted in parallel. For this purpose: (1) digestion was stopped at the end of the gastric phase by adjusting the pH to 7.0; and (2) digestion was carried out until the end of the intestinal phase and enzymes were inhibited by heat treatment (85 °C/15 min). As a blank control for the analysis, the sample was replaced with ultrapure water in a digestion experiment. The digestion experiments were carried out in triplicate.

### 2.3 Reversed-Phase High-Performance Liquid Chromatography (RP-HPLC)

Reversed-phase high-performance liquid chromatography (RP-HPLC) of the gastric digests was performed using a Shimadzu HPLC system (Snoqualmie, WA, USA) equipped with a diode array detector and a Biozen 3 μm Peptide PS-C18 column (150 × 4.6 mm, Phenomenex, Torrance, CA, USA). The operating conditions were as follows: column at room temperature (25 °C); detection at 214 nm; flow rate of 1 mL/min; injection volume of 20 μL; mobile phase A, 0.04% trifluoroacetic acid (TFA) (Sigma-Aldrich Co., St. Louis, MO, USA) in ultrapure water; mobile phase B, 0.03% TFA in acetonitrile (J.T.Baker-Avantor, Xalostoc, Mexico); and both solvents filtered through a 0.45 μm membrane. A linear gradient of solvent B in part A was applied. Initially, the column was equilibrated with solvent A; solvent B was increased from 0 to 50% over 45 min, then from 50 to 70% until 50 min, holding at 70% B for 5 min. The gradient was then returned to the initial condition by decreasing solvent B from 70 to 0% over 5 min, followed by 10 min at 100% solvent A. The total running time was 70 min. The digests were diluted in solvent A (400 μL/mL) and filtered through a 0.22 μm membrane (GVS Life Sciences, Sanford, ME, USA). Samples were analyzed in triplicate. Chromatograms were generated using OriginPro 9.0 software (OriginLab Corp., Massachusetts, USA) from the raw data obtained from the chromatograph as well as for the calculation of the area under the curve (AUC).

### 2.4 Peptide Profiling by Liquid Chromatography–Tandem Mass Spectrometry (LC-MS/MS)

Initially, the digests were centrifuged at 3000 × g for 30 min at 4 °C using an Allegra 64R centrifuge (Beckman Coulter, Indianapolis, IN, USA). The supernatants were filtered through 0.22 μm polyvinylidene membranes, followed by ultrafiltration using a 10 kDa molecular weight cutoff filter (5000 × g at 4 °C). The filtrates were then subjected to peptide microextraction by solid-phase StageTip (stop-and-go extraction tips), as described by Rappsilber; Mann; Ishihama,^24^ with modifications by Liu; Pischetsrieder,^25^ and subsequently analyzed for peptide profiles by liquid chromatography coupled to tandem mass spectrometry (LC–MS/MS).

An aliquot of the extracts obtained (∼0.2 μg) was analyzed on a mass spectrometer Orbitrap Velos (Thermo Fisher Scientific, Waltham, MA, USA) connected to the EASY-nLC system (Proxeon Biosystem, West Palm Beach, FL, USA) through a Proxeon nanoelectrospray ion source at the Biosciences National Laboratory (LNBio) of the Brazilian National Center for Research in Energy and Materials (CNPEM). Separation of the peptides was achieved by a 2–30% acetonitrile gradient in 0.1% formic acid using an analytical column PicoFrit Column (20 cm x ID75 μm, 5 μm particle size, New Objective) at a flow rate of 300 nL/min over 35 min. The nanoelectrospray voltage was set to 2.2 kV and the source temperature was set to 275 °C. Data were acquired in the data-dependent acquisition mode (DDA). The full scan MS spectra (*m*/*z* 300–1600) were acquired in the Orbitrap at a resolution of *r* = 60,000 with a target value of 1e6. The 20 most intense peptide ions (charge ≥ 1) were sequentially isolated to a target value of 5,000 and fragmented in the linear ion trap using low-energy CID (normalized collision energy of 35%). The signal threshold for triggering an MS/MS was 1,000 counts. Dynamic exclusion was enabled with an exclusion size list of 500, an exclusion duration of 60 s, and a repeat count of 1. Activation was performed with *q* = 0.25 and 10 ms activation time.

After data acquisition, the spectra were analyzed using PEAKS Studio 12 software (Bioinformatics Solutions Inc., Waterloo, ON, Canada) against the UniProt database with *Bos taurus* selected as the taxonomy. Both precursor mass error tolerance (monoisotopic) and fragment ion tolerance were set to 0.5 Da. The database search was configured with a semispecific digest mode, with the enzymes specified by each sample type and three maximum missed cleavages. To simulate the sequential digestive phases, a multiple-enzyme strategy was employed: pepsin for gastric digests and both trypsin and chymotrypsin for intestinal digests. Although variable modifications were considered during peptide identification, downstream analyses were restricted to the corresponding unmodified sequences. The peptide score calculated by PEAKS of −10lgP ≥ 20 was applied, corresponding to a p-value of 0.01 or lower, indicating a statistically confident peptide-spectrum match. False discovery rate (FDR) estimation was enabled. Given the study’s main interest in comparing the peptide profiles of the A1 and A2 variants of β-CN, only peptides derived from the precursor protein β-CN (UniProt ID: P02666|CASB_BOVIN, accessed on January 17, 2025) were extracted for further analysis.

### 2.5 Analysis of Identified Peptides and Evaluation of Enzymatic Cleavage Sites

Based on the raw data obtained with the PEAKS software, peptides derived from the β-CN A2 sequences were extracted and subjected to two filters. First, only peptides detected in all three samples (i.e., triplicate digestions) were selected. Second, peptides identified in the blank digests were excluded from the resulting list to eliminate nonspecific interferences. The final set of peptides, considered representative of the protein hydrolysis induced by digestion, was used for subsequent analyses.

After peptide selection, Venn diagrams were generated using Python (ver. 3.12) with the *matplotlib_venn* library, allowing visualization of the overlap among the analyzed sets. The heat map was constructed using Python (version 3.12) for fragment reading and mapping of the corresponding positions within the β-CN sequence. Data were organized in Microsoft Excel (version 2504), and the final figure was generated using the online platform Flourish (https://flourish.studio).

An in-silico evaluation was performed using the Peptide Cutter bioinformatics tool^26^ to identify the cleavage sites of the digestive enzymes pepsin, trypsin, and chymotrypsin in the β-CN sequence, with the aim of assisting in the understanding of the hydrolysis process and the peptides generated.

### 2.6 Statistical Analysis

The comparison between the means of the two products was performed using a two-tailed Student’s *t* test, at 5% significance level (*P* < 0.05). Results were expressed as mean ± standard deviation. Statistical analyses were carried out using Microsoft Excel, version 2504.

## 3 Results and Discussion

### 3.1 Physicochemical Composition of A1/A2 and A2/A2 Milks

The physicochemical composition of A1/A2 and A2/A2 milk samples was as follows (mean ± SD, expressed as % w/v): protein, 3.31 ± 0.25 and 3.41 ± 0.03; fat, 1.09 ± 0.01 and 2.07 ± 0.003; total solids, 10.13 ± 0.02 and 11.07 ± 0.02; ash, 0.75 ± 0.01 and 0.76 ± 0.01; and lactose, 4.98 ± 0.26 and 4.83 ± 0.02, respectively. No significant differences were observed in the protein, lactose, and ash content between the milk types. However, the fat and total solids content showed statistically significant differences (*p* < 0.05). Although the fat content differed significantly between the milks, with A2/A2 milk containing approximately 1% more fat, both samples fall within the classification of semiskimmed milk according to Brazilian regulations.^27^ The significant difference in total solids can likely be attributed to the variation in fat content. Similar results have been reported by Daniloski et al.,^28^ Nguyen et al.,^6^ and Costa-Santos et al.^29^ who observed similarity in the composition of milks containing β-CN A1 and A2.

### 3.2 Production of Yogurts

The acidification behavior of A1/A2 and A2/A2 milks during fermentation is shown in Figure 1A. The average time required to reach pH 4.8 did not differ between A1/A2 (240 ± 0.0 min) and A2/A2 (250 ± 8.66 min) milks (*P* = 0.1835), and both milks exhibited similar acidification profiles (Figure 1A).

**Figure 1:**
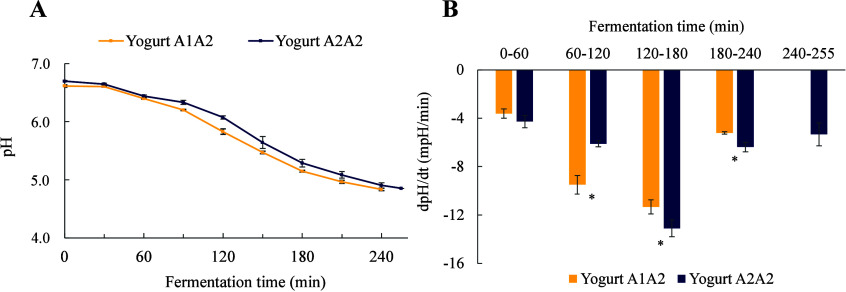
Acidification profile of A1/A2 and A2/A2 milks during fermentation. (A) pH curve and (B) rate of acidification. Mean values of three replicates (*n* = 3). The error bars represent the standard deviation of the mean. *Indicates significant difference (*P* < 0,05) between milks.

During the first 60 min of incubation, no differences in acidification rate were observed between A1/A2 and A2/A2 milks. However, between 60 and 120 min, the acidification rate of A1/A2 milk more than doubled and became significantly higher than that of A2/A2 milk (Figure 1B). This behavior may be related to the availability of nutrients necessary for the growth and metabolism of the microorganisms involved in fermentation (*Streptococcus thermophilus* and *Lactobacillus delbrueckii* subsp. *bulgaricus*). The associative growth of these microorganisms is a classical example of microbial cooperation. Initially, *L. bulgaricus*, which has a higher proteolytic activity, promotes the growth of *S. thermophilus* by producing small peptides and amino acids, especially valine. In turn, *S. thermophilus* stimulates the growth of *L. bulgaricus* by producing formic acid from pyruvate under anaerobic conditions and rapidly generating CO_2_. Considering this cooperative cycle, it is plausible that differences in protein conformation may facilitate or hinder protein hydrolysis and amino acid release, thereby affecting the rate of microbial metabolite production, particularly during the initial hours of fermentation. Between 120 and 180 min, the maximum acidification rate was observed for both milks, with the rate for A2/A2 milk being significantly higher than that for A1/A2 milk.^2^

### 3.3 Chromatographic Profile (RP-HPLC) of the Gastric Digests of Yogurts

Figure 2 shows the reversed-phase high-performance liquid chromatography (RP-HPLC) results of the A1/A2 and A2/A2 yogurts after gastric digestion. The chromatograms of gastrointestinal digests were initially evaluated. However, as they showed results similar to the digestion blanks, further analysis was not pursued. The peptide profiles of the two digests (A1/A2 and A2/A2) were similar (Figure 2A). To compare them, the area under the curve was calculated in 5 min elution intervals, indicating the relative concentration of peptides eluted in each interval (Figure 2B).

**Figure 2:**
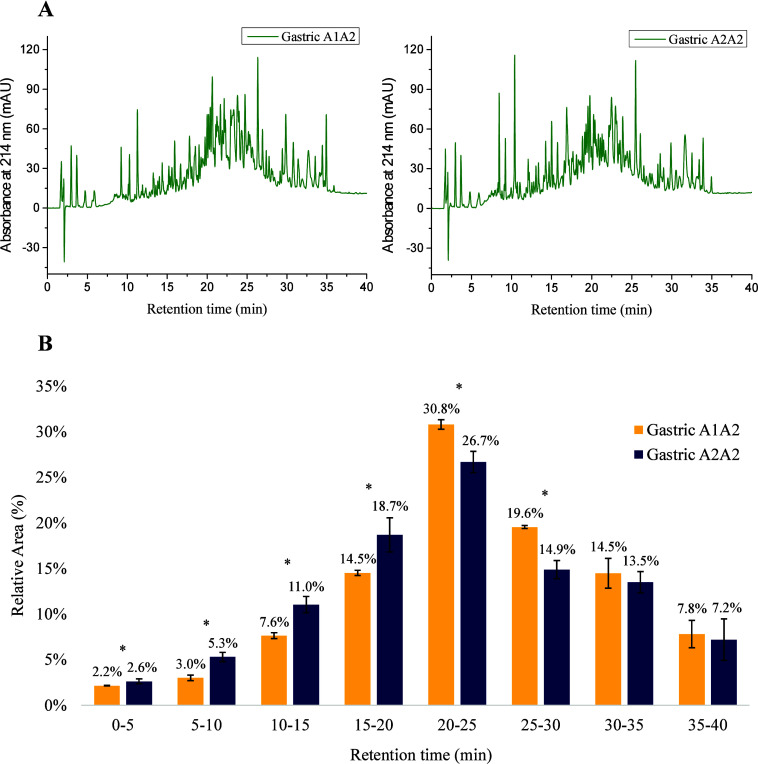
Chromatographic profiles and distributions of the gastric digests of A1/A2 and A2/A2 yogurts. (A) Peptide profiles obtained by reversed-phase high-performance liquid chromatography (RP-HPLC) at 214 nm absorbance for A1/A2 gastric digest (left) and A2/A2 gastric digest (right). (B) Relative area calculated for 5 min elution intervals based on the total area under the curve (AUC). Error bars represent standard deviation. *Indicates significant difference (*P* < 0.05).

During the first 20 min of elution, the gastric digests of A2/A2 yogurt showed a higher relative concentration than those of A1/A2 yogurt, suggesting a distinct hydrolysis pattern with greater release of hydrophilic peptides during gastric digestion of A2/A2 yogurt. These differences in the pepsin hydrolysis patterns of A1/A2 and A2/A2 yogurts may be associated with alterations in the protein conformation. Considering that caseins are naturally rich in proline, the presence of an additional proline at position 67 of β-CN A2 favors the formation of polyproline II helices,^30^ which partially resembles the α-helix but is more extended and flexible.^31^ Such an alteration in protein secondary structure may impact casein micelle organization, which may have affected enzyme accessibility to the hydrolysis sites. Variations in micelle size and κ-CN content, for example, have already been correlated with the degree of hydrolysis by gastric enzymes.^32^ Importantly, these results should also be interpreted by considering the matrix effect during digestion. Unlike liquid milk, in which caseins precipitate and form clots in the stomach, yogurt is already structured as an acid-induced protein gel. Therefore, this finding may be associated with differences in the yogurt matrix formed during acid-induced coagulation. Several authors have reported differences, sometimes subtle, in the protein network structure between acid gels produced from A2/A2 milk and milk containing β-CN A1. Daniloski et al.,^33^ Nguyen et al.^6^ and Semerci et al.^34^ observed that yogurts containing exclusively β-CN A2 exhibited a more porous microstructure, with finer and less dense

protein strands, compared to yogurts made from milk containing β-CN A1. These differences were found to be most pronounced between homozygous genotypes A2/A2 and A1/A1. Consequently, a denser or less dense gel microstructure would be expected to influence enzyme accessibility and proteolysis during gastric digestion.

### 3.4 Peptide Profiling of the A1/A2 and A2/A2 yogurts and their gastric and gastrointestinal digests using LC-MS/MS

The numbers of β-CN-derived peptides identified by using the PEAKS software are presented in Table 1. In the subsequent analyses, only the peptides selected after applying the filters (described in section 2.5) were considered (Supporting Information), as represented in the Venn diagram (Figure 3).

**Figure 3:**
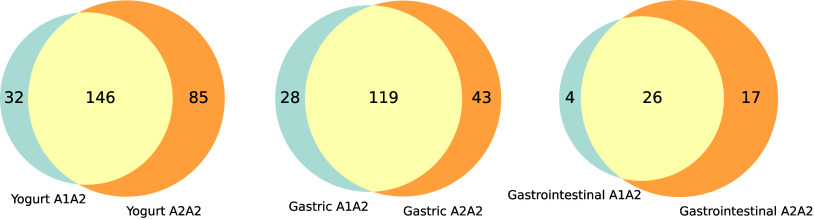
Number of peptides identified in yogurts and gastrointestinal digests after filtering. A2/A2 samples are represented by orange-colored areas, A1/A2 samples by blue areas, and the overlapping regions by light beige.

**Table 1:** Number of β-CN-Derived Peptides Detected in A1/A2 and A2/A2 Yogurts and Their Digests during the Gastric and Gastrointestinal Digestion Stages

	A1/A2	A2/A2
Total number of identified peptides		
Yogurt	407	470
Gastric digest	379	437
Gastrointestinal digest	79	101
Number of peptides in the three replicates (Filter 1)		
Yogurt	178	231
Gastric digest	178	194
Gastrointestinal digest	31	44
Number of peptides in the three replicates minus the peptides identified in the digestion blanks (Filter 2)		
Yogurt	178	231
Gastric digest	147	162
Gastrointestinal digest	30	43

The number of peptides selected in the yogurts, before and after gastrointestinal digestion, and the number of peptides shared between A1/A2 and A2/A2 milk samples are represented by Venn diagrams (Figure 3). Overall, a higher number of total and unique peptides were identified in A2/A2 yogurts both before and after digestion compared to A1/A2 yogurt. The 146 peptides shared between the yogurts corresponded to 82% and 63% of the total peptides identified in A1/A2 and A2/A2 yogurts, respectively. Approximately 40% of the peptides identified in A2/A2 yogurt were unique, i.e., absent in A1/A2 yogurt. In the yogurts, peptides were released by proteolytic enzymes produced by both the lactic culture and endogenous milk enzymes, such as plasmin.^35,36^

After digestion, a decrease in the number of identified peptides was observed, particularly after gastrointestinal digestion, which may be attributed to the extensive hydrolysis promoted by intestinal enzymes, which degrades peptides into fragments too small to be detected. Similar observations have been reported by Jin et al.^37^ regarding the hydrolysis of β-CN and κ-CN fractions following gastrointestinal digestion of yogurt. In the diagram representing the peptides released during this stage (Figure 3), both a higher total number (43) and a greater number of unique peptides (17) were observed in the A2/A2 set. The shared peptides represented 60.5% of the total in the A2/A2 set, while in the A1/A2 sample they accounted for 86.7%. These data highlight a greater peptide diversity in the A2/A2 digest, with a proportion of unique peptides of 39.5%, which is three times higher than that observed in the A1/A2 digest (13.3%).

Using the selected peptides, a heat map was generated (Figure 4), showing the positions of the fragments along the β-CN sequence. The color intensity corresponds to the frequency with which each amino acid was identified in the peptides along the β-CN sequence, ranging from 0 (white) to the maximum number of peptides (dark red). The values were normalized within each group: yogurt, gastric digest, and gastrointestinal digest.

**Figure 4:**
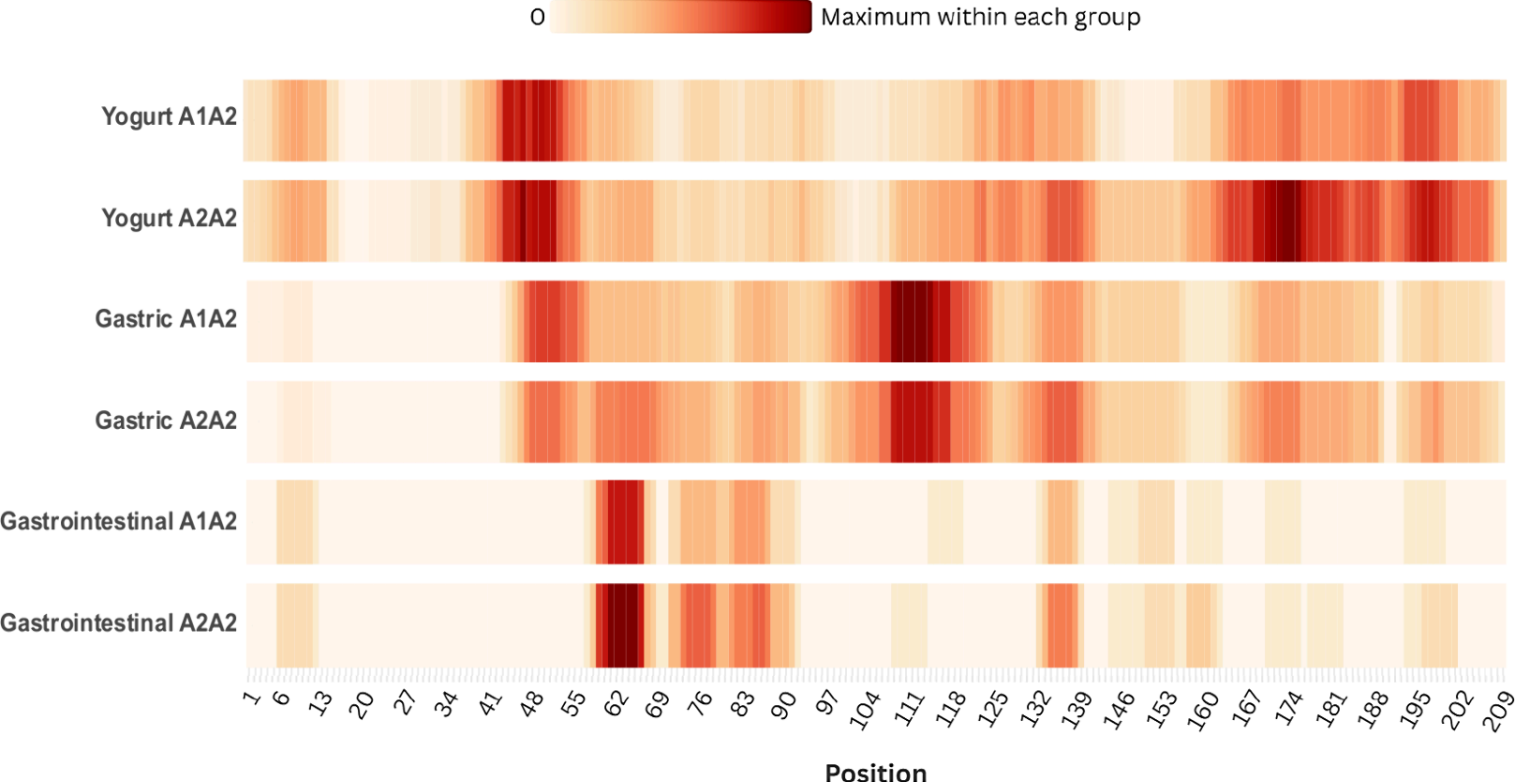
Heat map of peptides derived from β-CN. The representation indicates the position within the 209 amino acids of β-CN from which the peptides originated. The color gradient reflects the frequency at which each amino acid was identified in the peptides, ranging from 0 peptides (white) to the maximum number of peptides (dark red), normalized within each of the three groups (yogurt, gastric digest, and gastrointestinal digest).

For both yogurts, the highest frequency of peptide release was observed in the region of the protein between the amino acids D_43_ and Q_54_, as well as in the C-terminal

region, where the distribution was broader along the amino acid sequence. However, in this region, the color intensity was higher for A2/A2 yogurt, indicating a greater release of peptides originating from the end of the β-CN sequence. The results also suggest differences in the most frequent hydrolysis sites, such as the S_122_–L_139_ region, where a more intense release of peptides was observed in A2/A2 yogurt.

After yogurt digestion, a decrease in the number of peptides derived from the N-terminal region of both β-CN variants was observed. This result may be related to the intense proteolysis in this region, which contains multiple cleavage sites for pepsin (7 sites from E_2_ to Q_34_), trypsin (12 sites from R_1_ to I_49_), and chymotrypsin (11 sites from L_3_ to A_53_). Asledottir et al.^18^ and Schmelzer et al.^38^ also reported a lower number of peptides released from the N-terminal region of these variants. According to Asledottir et al.,^18^ this may be associated with the phosphorylation of serine residues in peptides originating from this region, which may reduce ionization efficiency in the ESI/MS source, thereby leading to low peptide detection.

In the present study, fragment YPFPGPI (Y_60_–I_66_), corresponding to the β-casomorphin-7 (β-CM7) peptide, was also identified after gastrointestinal digestion of both yogurts. Several studies have reported the identification of the opioid peptide β-CM7 in gastrointestinal digests of milk and cheese containing both β-CN A1 and A2, with some reporting higher concentrations released from A1 samples.^17,39^ However, in contrast to these reports, in the present study this peptide was detected with greater intensity in the A2/A2 yogurt digest (1.57 × 10^7^ AUC) compared to the A1/A2 yogurt digest (8.26 × 10^6^ AUC). This unexpected result may be attributed to factors related to differences in the yogurt matrix or additional degradation of the A1/A2 yogurt digest during the intestinal phase. Variations in protein network formation during acid-induced coagulation, as reported by Daniloski et al.,^33^ who observed larger micelles in A2/A2 yogurt, may influence the extent of protein hydrolysis during gastrointestinal digestion.^17^ This mechanism suggests that micelles formed by β-CN A2 may undergo more extensive hydrolysis, thereby promoting a greater release of peptides from A2/A2 yogurt. Although the literature does not report higher concentrations of β-CM7 in A2/A2 samples, some studies have indicated small differences in the concentration of this peptide between samples containing β-CN A1 and those composed exclusively of β-CN A2.^19,40,41^ Another hypothesis considered is the degradation of β-CM7 into proteolytic fragments by digestive enzymes during the digestion of A1/A2 yogurt, resulting in a reduced detection of this opioid peptide.

Another opioid peptide, β-casomorphin-9 (β-CM9), corresponding to the fragment YPFPGPIPN (Y_60_–N_68_), was also detected in both yogurt samples but not after digestion. In a recent study, Aitchison et al.^21^ reported lower amounts of β-CM7 after gastrointestinal digestion of β-CN A2 milk compared to β-CN A1 milk. On the other hand, β-CM9 was detected with greater intensity in the digested sample containing β-CN A2, while its amount in the β-CN A1 sample was considered negligible.

The fragment VYPFPGPIPN (V_59_–N_68_) was identified in the yogurts, as well as in their gastric and gastrointestinal digests, suggesting resistance to gastrointestinal digestion. This peptide contains the β-CM9 sequence, with only one additional amino acid (V_59_). β-CM9 may be further released through the action of brush-border peptidases.^37,42^ The V-β-CM9 fragment was also identified by Asledottir et al.^18^ after gastrointestinal digestion of purified β-CN A2. This peptide is considered bioactive, exhibiting angiotensin I-converting enzyme (ACE) inhibitory activity, as well as antioxidant properties.^43,44^ Cattaneo et al.^34^ mentioned that the release of BCM may be influenced by different conditions during gastrointestinal digestion, particularly by specific parameters of the *in vitro* digestion method employed, the digestion time, and the enzyme/protein ratio in the intestinal phase. Therefore, the identification of embedded BCM-containing peptides may lead to the generation of opioid peptides (such as β-CM7), depending on gastrointestinal conditions.^45^

Additionally, the fragments L_133_–L_139_ and V_59_–Q_72_ were identified in A2/A2 yogurt both before and after gastric and gastrointestinal digestion, therefore suggesting resistance to digestion.

Other identified peptides contained casomorphin sequences embedded within their structures. Most of these peptides have valine or leucine residues at their N-termini, which may indicate a tendency for hydrolysis between amino acids L_58_–V_59_ and S_57_–L_58_. These positions correspond to pepsin cleavage sites; in the case of the L_58_–V_59_ bond, cleavage by chymotrypsin may also occur.^26^

In the gastric digests, the highest number of released peptides was concentrated in the central region of β-CN (A_101_–Q_123_), with greater intensity in the A1/A2 digest, particularly in the E_108_–Y_114_ region. Similarly, Schmelzer et al.^33^ observed several preferential cleavage sites for pepsin between amino acids 124–130 and in regions closer to the C-terminal of the protein. Both gastric digests released peptides in the Q_46_–T_55_ region; however, in the A2/A2 yogurt gastric digest, other regions of peptide release were more prominent, showing higher color intensity compared to that of the A1/A2 digest (Figure 4). These findings suggest that while the peptides released from A1/A2 yogurt in the stomach are mainly derived from the central region of the protein, between amino acids 100–120, the peptides released during gastric digestion of A2/A2 yogurt originate from a broader region of β-CN, with less concentration in the central portion.

In the gastrointestinal digests of A1/A2 and A2/A2 yogurts, the greatest peptide release occurred between amino acids V_59_–I_66_, a region where BCMs can be released. The L_58_–V_59_ bond is susceptible to hydrolysis by pepsin and chymotrypsin. After cleavage of this bond, the enzyme responsible for releasing BCMs, through cleavage between V_59_–Y_60_, is leucine aminopeptidase (LAP).^14,20^ Therefore, the absence of peptides with Y_60_ at the N-terminus observed in this study may be related to low LAP activity at this cleavage site.

Other regions were also prominent in both gastrointestinal digests but with greater intensity in the A2/A2 digest (P_71_–T_78_, P_81_–F_87_, and H_134_–P_138_). Additionally, regions with the release of peptides unique to either A1/A2 or A2/A2 yogurts were observed. In the Y_114_–F_119_ region, peptide release occurred during the gastrointestinal digestion of A1/A2 yogurt, while this region was of little relevance in A2/A2 yogurt. In this segment, peptide YPVEPF (Y_114_–F_119_) was released. Similarly, the peptides EMPFPK (E_108_–K_113_) and AVPYPQ (A_177_–Q_182_) were fragments released exclusively in the A2/A2 gastrointestinal digest.

Overall, the differences in peptide abundance observed between A1/A2 and A2/A2 samples reflect variations in protein hydrolysis during yogurt processing and throughout the different stages of digestion, associated with the distinct β-CN genetic variants. Notably, these differences were distributed along the amino acid sequence and were not confined to position 67, the specific site of variation between A1 and A2 β-casein. This suggests that a single amino acid substitution may influence the susceptibility of multiple regions of the β-casein molecule to enzymatic hydrolysis.

In summary, the data demonstrate that during the first 60 min, A1/A2 and A2/A2 milks exhibited similar acidification rates. Subsequently, differences in the fermentation pattern suggest that the protein structure may have influenced amino acid release and, consequently, microbial metabolism. The differences in the peptide profiles of yogurts produced with the β-CN genetic variants are not limited to the region of amino acid 67, the distinguishing point between β-CN A1 and A2, but extend to other regions of the peptide chain. These differences were observed both in the yogurts prior to digestion and after the *in vitro* simulation of gastrointestinal digestion, confirming the impact of β-CN genetic variants on the peptides released throughout the digestive process. Furthermore, β-casomorphin-7 was identified in both A1/A2 and A2/A2 digests, reinforcing that the release of this bioactive peptide occurs regardless of the β-CN genetic variant used in yogurt production.

## Supplementary Material



## Notes

Peptides selected for analysis after application of filters (xlsx). (XLSX)

## †

Cargill Brasil, São Paulo, Brazil
